# The interaction effect between physical and cultural leisure activities on the subsequent decline of instrumental ADL: the Fujiwara-kyo study

**DOI:** 10.1186/s12199-019-0826-4

**Published:** 2019-12-01

**Authors:** Masayo Komatsu, Kenji Obayashi, Kimiko Tomioka, Masayuki Morikawa, Noriko Jojima, Nozomi Okamoto, Norio Kurumatani, Keigo Saeki

**Affiliations:** 1 Department of Epidemiology, Nara Medical University School of Medicine, 840 Shijocho, Kashiharashi, Nara, 634-8521 Japan; 2 Department of Public Health Nursing, Nara Medical University School of Medicine, Nara, Japan; 3 Nara Prefectural Health Research Center, Nara Medical University School of Medicine, Nara, Japan; 4 Mie Prefectural Mental Care Center, Mie, Japan; 5 Department of School Psychology, Development Science & Health Education, Hyogo University of Teacher Education, Hyogo, Japan

**Keywords:** IADL, Leisure physical activity, Cultural leisure activity, Interaction

## Abstract

**Background:**

Maintenance of instrumental activities of daily living (IADL) and social role (SR) is crucial to keep independent life because the decline in SR and IADL was a significant predictor of dependence in basic ADL in later. The independent effect of physical and cultural leisure activities and their effect modification on the IADL remains unknown.

**Methods:**

We prospectively observed 3241 elderly with intact IADL at baseline for 5 years. Higher level functional capacity such as IADL and SR was assessed using the Tokyo Metropolitan Institute of Gerontology Index of competence (TMIG index).

**Results:**

The mean age of the participants was 72.3 years (standard deviation 5.1), and 46.9% were male, and 90.9% of them received a follow-up assessment. Of the participants, 10.4% developed an IADL decline. Engagement in leisure physical activity was associated with a significantly lower risk of IADL decline (adjusted risk ratio, 0.73; 95% confidence interval [CI], 0.60 to 0.89), and cultural leisure activity was also associated with lower risk of IADL decline (adjusted risk ratio, 0.77; 95% CI, 0.63 to 0.95) independent of potential confounders. We also found significant and positive interaction between physical and cultural leisure activities at risk for IADL decline (*P* = 0.024) and SR decline (*P* = 0.004).

**Conclusions:**

We found an independent association of physical and cultural leisure activities with a lower risk for functional decline in IADL and SR with positive interaction. Combined engagement in physical and cultural activities may effectively prevent from IADL decline and SR decline.

## Introduction

To sustain an independent life with a satisfying quality of life, maintenance of function of the activities of daily living (ADL) is essential. In addition to the basic ADL (eating, bathing, and dressing), the importance of the instrumental ADL (IADL) is associated with the subsequent decline of basic ADL [1]. The Tokyo Metropolitan Institute of Gerontology Index of Competence (TMIG index) is a commonly used scale of higher level functional capacity [2], validated among Japanese participants, [3] which include three components such as IADL, intellectual activity (IA), and social role (SR). A longitudinal study using the TMIG index showed a hierarchical association of these components and basic ADL. The appearance of a decline in SR and IA among the elderly was a significant predictor of IADL decline in later and followed by dependence in basic ADL [4–6]. Furthermore, higher ADL is associated with lower mortality, [7, 8] and ADL decline is associated with higher medical cost and social care [9, 10].

Engagement in leisure activity shows benefit in terms of prevention from cognitive decline [11–13] and dementia [14–19], which is one of the most critical risk factors of ADL decline [20, 21]. Leisure activity mainly consists of physical activity and cultural activity. Higher physical activity is associated with a lower risk of decline in basic ADL and IADL [22–24]. The kind specific benefit of leisure activities to keep IADL remains unknown. Monma et al. found that engagement in leisure physical activity and cultural leisure activity are independently associated with a lower risk of basic ADL decline [25], but information about the independent effect of each kind of leisure activity on IADL is lacking. If the combined engagement in physical and cultural leisure activities strengthens the effect on the prevention from functional decline, we can recommend participation in both kinds of leisure activity. However, the effect modification by each type of leisure activity also remains unknown.

The purpose of the present study is to investigate the independent effect of leisure physical activity and cultural leisure activity on the subsequent functional decline of IADL, IA, and SR, and to access the interaction between physical and cultural leisure activities.

## Methods

### Participants

The Fujiwara-kyo cohort study enrolled 4427 community-dwelling elderly aged ≥ 65 years on a volunteer basis in four cities in Nara prefecture. The inclusion criteria for the study were the ability to walk without assistance from another person, to respond to self-reported information, and to provide written informed consent. The baseline examination was conducted from 2007 to 2008 [26]. All participants provided written informed consent at baseline. Eligibility criteria for the present cohort study were living in their own homes and able to walk independently. In the baseline examination, we assessed the higher level functional capacity in IADL, IA, and SR and the basic characteristics of the participants. After 5 years, we conducted a follow-up examination of higher level functional capacity. To investigate the longitudinal association between leisure activity at baseline and incident of decline in higher level functional capacity during 5 years, we included all participants with higher level functional capacity at baseline, and who completed the follow-up assessment. The Nara Medical University ethics committee approved the study protocol.

### Assessment of IADL decline, SR decline, and IA decline

We assessed IADL, SR, and IA using the TMIG Index consisting of 13 items. The response to each item was designed as “able to do” or “unable,” and scored 1 for able and 0 for unable. Thus, the maximum score of the TMIG index is 13 points. The TMIG Index has three subscales such as IADL (5 points: using bus or train, shopping, meal preparation, paying bill, and banking), IA: intellectual activity (4 points: filling out forms of pension, reading newspaper, reading books, and interest in new story and program about health), and SR: social role (4 points: visiting friends, being called on advise, visiting sick friends, and conversation with young people) [3]. We regarded participants with a low score in IADL (≤ 4 points), IA (≤ 2 points), and SR (≤ 2 points) as declined function, and we regarded participants with a high score in IADL (5 points), IA (3 or 4 points), and SR (3 or 4 points) as intact functional capacity. Fujiwara et al. assessed the validation of the threshold of the ADL decline based on the test-retest variability in the score of IADL, IA, and SR within one month [27].

### Physical and cultural leisure activities

Using self-administered questionnaire, we asked participants about participation in four kinds of leisure activity consist of physical activity (walking, exercise, jogging, playing ground golf, and gate ball), cognitive activity (reading books, learning foreign language, making poem, and playing board game or card), music activity (playing instrument, singing, and dancing), art activity (drawing pictures, taking a photograph, and making art craft). We categorized cognitive activity, music activity, and art activity into a cultural leisure activity. We asked participants about the frequency of participation using six following options such as “daily,” “several days per week,” “once a week,” “monthly,” “yearly,” and “never.” We regarded activities with “once a week” or more frequent as engagement in the present analysis [28].

### Other variables

Using a self-administered questionnaire, we asked about age, gender, the history of cancer, stroke, and myocardial infarction, and treatment of diabetes and hypertension, smoking and drinking habit, education history, and the number of a household member. We asked social participation other than leisure activity such as political activity, business activity, volunteer activity, consumer activity, religious activity, and a neighborhood association. We asked depressive symptoms using the short version of the Geriatric Depression Scale (GDS-15) [29]. Cognitive functions were examined using the Mini-Mental State Examination (MMSE) by trained research staffs. We measured height and body weight, and the body mass index was calculated as body weight (kg) divided by square of height (m).

### Statistical analysis

We compared mean values and proportions between the two groups using the *t* test and chi-square tests, respectively. The number of missing data was 2 in the history of stroke, 2 in the history of myocardial infarction, 7 in smoking habit, 7 in living alone, 15 in BMI, 24 in cognitive function, 26 in education length, 40 in social participation, 41 in ethanol intake, and 81 in depressive symptoms. For missing data, we conducted multiple imputation based on the multivariable logistic regression model which include age, gender, low BMI, high BMI, smoking habit, ethanol intake, comorbidity such as cancer, stroke, myocardial infarction, diabetes and hypertension, education length, living alone, depressive symptoms, cognitive impairment, and social participation other than leisure activity. Crude risk ratio and 95% confidence interval (CI) was estimated using a generalized estimation equation of a Poisson log-linear regression models which include the incidence of the functional decline in IADL or IA, and SR as a dependent variable and engagement in a leisure physical activity or cultural leisure activity as an independent variable (Table 2). Adjusted risk ratio and 95% CI was estimated using a multivariable model which included incidence of functional decline in IADL or IA or SR as a dependent variable and the independent variables consist of engagement in leisure physical activity and cultural leisure activity, score of baseline function of IADL or IA or SR, potential confounding variables such as age (< 73 years [median age at baseline] vs. ≥ 73 years), gender (male vs. female), low BMI (< 18.5, vs.18.5 to 25), high BMI (≥ 25, vs. 18.5 to 25) , smoking habit (yes vs. no), ethanol intake (yes vs. no), comorbidity such as cancer (yes vs. no), cardiovascular disease (yes vs. no), diabetes (yes vs. no), and hypertension (yes vs. no), education length (< 16 years vs. ≥ 16 year), living alone (yes vs. no), depressive symptoms (GDS-15 score ≥ 6 vs. < 6), cognitive impairment (MMSE ≤ 23 vs. > 23), and engagement in social activity other than leisure activity (yes vs. no). In the model which includes SR decline as the dependent variable, we excluded the term “social activity” from the independent variables. To access the interaction between leisure physical activity and cultural leisure activity, we constructed an interaction term (engagement in leisure physical activity and cultural leisure activity vs. others). We estimated *P* value for interaction based on the multivariable model which include dependent variable (incidence of functional decline) and independent variables simultaneously including leisure physical activity, cultural leisure activity, interaction term, all confounding variables and the number of leisure activity (< 2 vs. ≥ 2) (Fig. 2). Furthermore, we categorized all participants into four groups such as group 1: leisure physical activity (–) and cultural leisure activity (–), group 2: leisure physical activity (+) and cultural leisure activity (–), group 3: leisure physical activity (–) and cultural leisure activity (+), group 4: leisure physical activity (+) and cultural leisure activity (+). We estimated risk ratios and 95% CI refer to group 1 after adjustment for all confounding factors (Fig. 2). As a sensitivity analysis, we constructed a model which excluded the term “cognitive impairment” from the independent variables. We conducted a sub-analysis based on the data who completed follow-up assessment of cognitive impairment using MMSE and estimated relative risk among subjects engaged in leisure physical activity and cultural leisure activity adjusted for cognitive impairment at follow-up. We considered a two-sided *P* value less than 0.05 to be significant. All statistical analyses were conducted using the SPSS ver. 24.

## Results

**Fig. 1:**
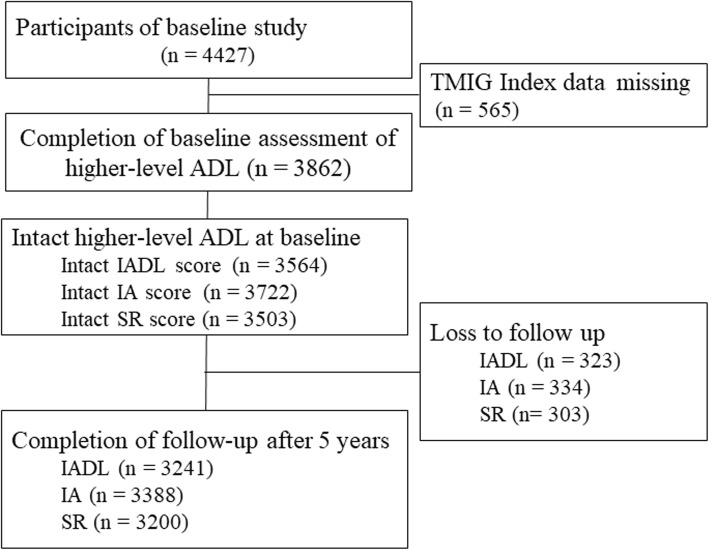
Flow of participants. IADL, instrumental activities of daily living; IA, intellectual activity; SR, social role

Of all 4427 participants of the baseline study, we assessed the higher level ADL using the TMIG index among 3862 participants, and the number of participants with the intact function was 3564 in IADL, 3722 in IA, and 3503 in SR. Of all participants with intact function at baseline, the proportion who completed follow-up assessment was 90.9% (*n* = 3241) in IADL, 91.0% (*n* = 3388) in IA, and 91.4% (*n* = 3200) in SR, respectively (Fig. 1). Compared with participants who completed follow-up assessment, those who were lost to follow-up was associated with higher age, female, non-drinker, stroke, cognitive impairment, depressive symptoms, and low education status (Additional file 1: Table S1). The incidence of IADL decline was associated with some characteristics at baseline such as higher age, male gender, lean body mass, the presence of smoking history, cancer and hypertension, cognitive impairment, depressive symptoms, lower education (Table 1). The risk of functional decline in IADL, IA, and SR in 5 years was 0.104, 0.079, and 0.104, respectively.

**Table 1:** Baseline characteristics by the incident of IADL decline during 5 years

	Intact IADL	IADL decline	*P* value
	*n* = 2905	*n* = 336	
Age, mean (SD)	71.7 (4.7)	75.7 (6.0)	< 0.001
Men, *n* (%)	1343 (46.2)	194 (57.7)	< 0.001
Obesity (BMI > 25), *n* (%)	631 (21.8)	67 (20.0)	0.45
Lean (BMI < 18.5), *n* (%)	169 (5.8)	38 (11.3)	< 0.001
Never smoker, *n* (%)	1786 (61.6)	169 (50.4)	< 0.001
Non-drinker, *n* (%)	1801 (62.8)	204 (61.4)	0.63
Comorbidity, *n* (%)			
Cancer	265 (9.1)	51 (15.2)	< 0.001
Stroke	142 (4.9)	21 (6.3)	0.28
Myocardial infarction	68 (2.3)	8 (2.4)	0.97
Diabetes	286 (9.9)	44 (13.1)	0.06
Hypertension	1104 (38.1)	148 (44.0)	0.034
Cognitive impairment, *n* (%)^*^	80 (2.8)	33 (9.9)	< 0.001
Depressive symptoms, *n* (%)^†^	362 (12.8)	63 (19.0)	0.002
Education (≥ 16 years), *n* (%)	2146 (74.4)	220 (66.3)	0.001
Social participation, *n* (%)^‡^	2435 (84.8)	283 (85.5)	0.74
Living alone, *n* (%)	41 (1.4)	2 (0.6)	0.22
Physical leisure activity, *n* (%)	1732 (59.6)	160 (47.6)	< 0.001
Cultural leisure activity, *n* (%)	1506 (51.8)	140 (41.7)	< 0.001
Both leisure activity, *n* (%)^⁋^	991 (34.1)	69 (20.5)	< 0.001

*BMI* body mass index (kg/m^2^), *IADL* instrumental activities of daily living, *SD* standard deviation

^*^MMSE (mini-mental scale examination ≤ 23)

^†^GDS-15 (geriatric depression scale ≥ 6)

^‡^Social participation other than leisure activities

^⁋^Participation to both physical leisure activity and cultural leisure activity

Engagement in leisure physical activity was associated with lower risk of IADL decline (risk ratio, 0.65; 95% CI, 0.53 to 0.79), IA decline (0.78, 0.62 to 0.98), and SR decline (0.78, 0.64 to 0.96) compared with those without it (Table 2). Engagement in cultural leisure activity was also associated with lower risk of IADL decline (risk ratio, 0.69; 95% CI, 0.56 to 0.85), IA decline (0.70, 0.56 to 0.89), and SR decline (0.73, 0.60 to 0.90) compared with those without it (Table 2).

**Table 2:** Risk for functional decline in 5 years by engagement in leisure activity

	Risk	Risk ratio (95% CI)	*P* value	Adjusted risk ratio (95% CI)	*P* value
IADL (*n* = 3241)					
Leisure physical activity					
Without (*n* = 1349)	0.130	1		1	
With (*n* = 1892)	0.085	0.65 (0.53 to 0.79)	< 0.001	0.73 (0.60 to 0.89)*	0.002
Cultural leisure activity					
Without (*n* = 1595)	0.123	1		1	
With (*n* = 1646)	0.085	0.69 (0.56 to 0.85)	< 0.001	0.77 (0.63 to 0.95)†	0.015
Intellectual activity (*n* = 3388)					
Leisure physical activity					
Without (*n* = 1400)	0.090	1		1	
With (*n* = 1988)	0.070	0.78 (0.62 to 0.98)	0.030	0.88 (0.70 to 1.11)*	0.29
Cultural leisure activity					
Without (*n* = 1671)	0.093	1		1	
With (*n* = 1717)	0.065	0.70 (0.56 to 0.89)	0.003	0.82 (0.64 to 1.04)†	0.10
Social role (*n* = 3200)					
Leisure physical activity					
Without (*n* = 1324)	0.119	1		1	
With (*n* = 1876)	0.093	0.78 (0.64 to 0.96)	0.018	0.87 (0.71 to 1.06)‡	0.17
Cultural leisure activity					
Without (*n* = 1569)	0.120	1		1	
With (*n* = 1631)	0.088	0.73 (0.60 to 0.90)	0.003	0.85 (0.70 to 1.05)⁋	0.14

*IADL* instrumental activities of daily living, *CI* confidence interval

^*^Adjusted for engagement in cultural leisure activity, baseline functional capacity (instrumental ADL or intellectual activity or social role), and potential confounders such as age, gender, BMI, smoking habit, ethanol intake, history of cancer cardiovascular disease, diabetes, hypertension, living alone, depressive symptoms, cognitive impairment, and participation to social activity

^†^Adjusted for engagement in leisure physical activity, baseline functional capacity (instrumental ADL or intellectual activity or social role), and all potential confounders

^‡^Adjusted for engagement in cultural leisure activity, baseline functional capacity, and potential confounders except for participation to social activity

^⁋^Adjusted for engagement in leisure physical activity, baseline functional capacity, and potential confounders except for participation to social activity

In multivariable analysis, engagement in leisure physical activity was significantly associated with a significantly lower risk for IADL decline (adjusted risk ratio, 0.73; 95% CI, 0.60 to 0.89) independent of baseline IADL, cultural leisure activity, and potential confounders such as age, gender, BMI, smoking habit, ethanol intake, past history of cancer cardiovascular disease, diabetes, hypertension, living alone, depressive symptoms, cognitive impairment, and participation to other social activity. Engagement in cultural leisure activity was also associated with a significantly lower risk of IADL decline (adjusted risk ratio, 0.77; 95% CI, 0.63 to 0.95) independent of leisure physical activity and potential confounders.

**Fig. 2:**
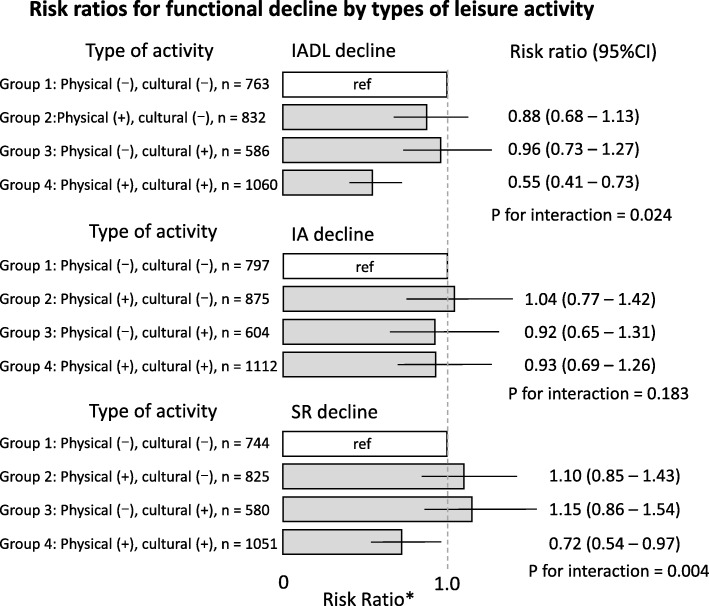
Risk ratios for functional decline by types of leisure activity. IADL, instrumental activities of daily living; IA, intellectual activity; SR, social role. Error bars show 95% confidence intervals of the risk ratio. *Adjusted for baseline functional capacity and potential confounders such as age, gender, body mass index, smoking habit, ethanol intake, history of cancer cardiovascular disease, diabetes, hypertension, living alone, depressive symptoms, cognitive impairment, and participation to social activity. The relative risk for SR decline was adjusted for all confounders except for participation in social activity. ^†^*P* values for interaction were based on the multivariable model which consist of the dependent variable (incidence of functional decline) and independent variables simultaneously including leisure physical activity, cultural leisure activity, interaction term, all confounding variables, and the number of leisure activity (< 2 vs. ≥ 2)

Compared with group 1 (without physical and cultural leisure activities), group 4 (engaged in both physical and cultural leisure activities) showed significantly lower risk for IADL decline (risk ratio, 0.55; 95% CI, 0.41 to 0.73) and SR decline (risk ratio, 0.72; 95% CI, 0.54 to 0.97) (Fig. 2). We found significant and positive interaction between leisure physical activity and cultural leisure activity in risk for IADL decline (P for interaction = 0.024) and SR decline (P for interaction = 0.004) independent of confounding factors and the number of leisure activity (Fig. 2).

Sensitivity analysis using multivariable model excluding cognitive impairment at baseline from the independent variables revealed consistent results that risk for IADL decline was significantly lower in subjects with leisure physical activity (adjusted risk ratio, 0.71; 95% CI, 0.59 to 0.88) and cultural leisure activity (adjusted risk ratio, 0.76; 95% CI, 0.61 to 0.94), and we found significant interaction between two kinds of leisure activity (*P* = 0.032).

Sub-analysis among participants who completed assessment of cognitive function at follow-up (*n* = 3081), even after further adjustment for cognitive impairment at follow-up, risk for IADL decline was significantly lower among subjects with leisure physical activity (adjusted risk ratio, 0.70; 95% CI, 0.55 to 0.89) and cultural leisure activity (adjusted risk ratio, 0.76; 95% CI, 0.59 to 0.97), but the interaction between them was not significant (P for interaction = 0.11).

## Discussions

Based on the population-based prospective cohort study among 3241 elderly participants for 5 years, engagement in leisure physical activity and cultural leisure activities were independently associated with a lower risk for decline in IADL. As far as we know, this is the first study to report significant and positive interaction between leisure physical activity and cultural leisure activity to a decreased risk of IADL decline.

The risk of decline in higher level functions such as IADL and SR, an early sign of basic ADL decline, was significantly lower among participants with engagement in physical and cultural leisure activities consistent with the previous studies about the risk of basic ADL decline. Monma et al. showed that leisure physical activity was independently associated with lower odds for basic ADL decline among men and women, and cultural leisure activity is associated with lower odds for basic ADL decline among women [25]. However, the effect of each kind of leisure activity on the IADL and higher functional capacity remained unknown. Here, we found that engagement in leisure physical activity and cultural leisure activity is independently associated with a lower risk of IADL decline.

Based on the risk for IADL decline among participants without engagement in leisure physical activity (0.130), and adjusted relative risk among participants with engagement in leisure physical activity (0.73), the number needed to prevent one person from IADL decline in 5 years is 28.4. The number needed to prevent from IADL decline by engagement in cultural leisure activity is 35.4. This clinical implication will be useful evidence to consider the policies to promote leisure activity to prolong healthy life expectancy. Besides, we found a significant interaction effect between physical and cultural leisure activities on ADL decline and SR decline. This finding suggests that a combination of physical and cultural leisure activities will enhance the benefit of each activity to prevent IADL decline and SR decline, and it may be useful to sustain an independent life [1, 4–6].

Mechanism of the interaction between physical and cultural leisure activities on functional decline remain unclear, but a bidirectional effect between higher engagement in physical activity and cultural leisure activity may partly explain the mechanism. Higher physical activity and exercise directly associated with sustained muscle strength, muscle volume and physical function, [30, 31] and it associated with a lower risk of obesity, diabetes, dyslipidemia, hypertension, and cardiovascular disease [32–36]. These benefits from physical activity may be associated with higher participation in cultural leisure activity. Participation in cultural leisure activity is associated with a lower risk of cognitive decline, [11, 13–16, 18, 19] and it may be related to maintenance of engagement in leisure physical activity. Also, recent studies about the beneficial effect of physical activity on brain structure and function may support the mechanism of the interaction. A randomized controlled study showed that aerobic exercise is associated with an increase of hippocampus volume accompanied by the higher secretion of BDNF (brain-derived neurotrophic factor) and improvements in spatial memory than the control group, [37] and epigenetic mechanism may regulate the beneficial effect [38]. Consistently, a longitudinal observational study among the elderly (*n* = 1375) also showed the onset of dementia was prolonged among participants to three kinds of leisure activity such as mental, social, and physical activities compared with those engaged in one or two kinds of activities [17]. Sub-analysis in the present study, adjustment for the cognitive impairment at follow-up attenuated the interaction between leisure physical activity and cultural leisure activity. This result was consistent with the hypothesis that the interaction of leisure activities for prevention from IADL decline was partly mediated by cognitive impairment.

The reason why we did not find a significant association between leisure activity and IA may be partly explained by a close correlation between cognitive function and IA decline. In the analysis in Table 2, we adjusted for cognitive impairment and IA at baseline as a confounding factor in the association between leisure activity and subsequent IA decline. However, cultural leisure activity may already effect on cognitive function and IA at baseline. Therefore, adjustment for cognitive impairment and IA at baseline may cause underestimation of the effect of leisure activity on subsequent IA decline. In an analysis excluding cognitive impairment and IA at baseline from the independent variables in the multivariable model of Table 2, we found a significant association between cultural leisure activity and subsequent IA decline (adjusted risk ratio, 0.77; 95% CI, 0.61 to 0.98; *P* value = 0.033).

The present study has several limitations. First, participants are not randomly sampled, and we excluded participants who lost to follow-up in the present study. The subjects lost to follow-up was associated with a higher prevalence of risk factors for IADL declines, such as higher age, stroke, cognitive impairment, depressive symptoms, and low education status. Sampling bias and lost to follow-up may reduce the generalizability of the results. However, the quality of the follow-up is acceptable because over 90% of the participants completed the follow-up assessment. Second, the primary exposure (engagement in leisure activity) and primary outcomes (functional capacity) in the present study were derived from the response to the self-administered questionnaire. Thus, the occurrence of misclassification may increase among subjects with cognitive impairment. However, the analysis adjusted for cognitive impairments at baseline revealed consistent results with the analysis without adjustment. Thirdly, we did not access the leisure physical activity in terms of the type and intensity, and we regarded leisure physical activities with “once a week” or more frequent as engagement. So, we may include the participants with an inadequate amount of physical activity according to the physical activity guidelines [39]. Fourthly, we investigated disease history and present illness using self-reported information and prescribed medication. Thus, the confounding effect of mild comorbidity without medication may remain. Finally, the present study was conducted in a single lesion in Japan, and the contents of leisure activity may depend on the cultural background. Further studies in multiple regions in several countries will be needed.

## Conclusions

We found engagement in leisure physical activity and cultural leisure activity was independently associated with a lower risk of IADL decline, and positive interaction between both kinds of leisure activity was significant in this prospective cohort study among the elderly.

## Supplementary information

**Additional file 1: Table S1.** Baseline characteristics by the completion of follow-up assessment of IADL

## Data Availability

The datasets analyzed during the current study are not publicly available due to ethical consideration but are available from the corresponding author on reasonable request.

## Abbreviations

IADL: Instrumental activities of daily living
IA: Intellectual activity
SR: Social role
TMIG index: Tokyo Metropolitan Institute of Gerontology Index of Competence
