# Supplementary material for: The interaction effect between physical and cultural leisure activities on the subsequent decline of instrumental ADL: the Fujiwara-kyo study
Source: Environ Health Prev Med. 2019 Dec 1;24:71. doi: 10.1186/s12199-019-0826-4 (PMC6886184; doi:10.1186/s12199-019-0826-4)
Supplement: Supplementary file 1 — Additional file 1: Table S1. Baseline characteristics by the completion of follow-up assessment of IADL [file 12199_2019_826_MOESM1_ESM.pdf]

Supplementary Table

Baseline characteristics by the completion of follow-up assessment of IADL

	followed up n = 3241	lost to follow n = 323	P-value
Age, mean (SD)	72.1 (5.0)	73.7 (5.5)	< 0.01
Men, n (%)	1537 (47.4)	130 (40.2)	0.01
BMI (≥25), n (%)	698 (21.6)	77 (23.9)	0.34
Never smoker, n (%)	1955 (60.5)	208 (65.0)	0.11
Non-drinker, n (%)	2005 (62.7)	226 (70.6)	< 0.01
Comorbidity, n (%)			
Cancer	316 (9.8)	33 (10.2)	0.79
Stroke	163 (5.0)	28 (8.7)	< 0.01
Myocardial infarction	76 (2.3)	8 (2.5)	0.88
Diabetes	330 (10.2)	33 (10.2)	0.99
Hypertension	1252 (38.7)	126 (39.0)	0.91
Cognitive impairment, n (%) *	113 (3.5)	22 (6.9)	< 0.01
Depressive symptoms, n (%) †	425 (13.5)	59 (18.8)	< 0.01
Education (≥16 y), n (%)	2366 (73.6)	208 (65.0)	< 0.01
Social participation n (%) ‡	2718 (84.9)	263 (82.2)	0.20
Living alone, n (%)	43 (1.3)	8 (2.5)	0.09

IADL: instrumental activities of daily living, BMI: Body Mass Index

[*] MMSE (Mini-Mental Scale Examination ≤ 23)

[†] GDS-15 (geriatric depression scale ≥ 6)

[‡] Social participation other than a leisure activity
